# Impact of Distance From the Hospital and Patient Transfer on Pediatric Testicular Torsion Outcomes

**DOI:** 10.7759/cureus.25284

**Published:** 2022-05-24

**Authors:** Lisa B Shields, Michael W Daniels, Dennis S Peppas, Eran Rosenberg

**Affiliations:** 1 Norton Neuroscience Institute, Norton Healthcare, Louisville, USA; 2 Department of Bioinformatics & Biostatistics, University of Louisville, Louisville, USA; 3 Pediatric Urology, Norton Healthcare, Louisville, USA

**Keywords:** orchiectomy, transfer, distance, emergency medicine, pediatrics, testicular torsion, pediatric urology

## Abstract

Background: Testicular torsion is the most common pediatric emergency that requires prompt diagnosis and surgical treatment to prevent testicular loss. Distance from the hospital where the patient will be undergoing treatment for testicular torsion and transfer from an outside facility are factors that may impact whether a testis is salvageable. We sought to determine whether these factors play a role in pediatric testicular torsion outcomes.

Materials and Methods: We identified males aged 1-18 years with testicular torsion between January 1, 2015 and December 31, 2020. The patients’ distance from our hospital and whether they were transferred from an outlying hospital were a particular focus.

Results: The number of miles from our hospital and transfer from an outlying hospital were not significantly different between boys who underwent an orchiectomy versus an orchiopexy (p=0.258 and p=0.574, respectively). The number of miles from our hospital was negatively correlated to age at surgery (rho=-0.22, p=0.01). Significantly (p<0.001) more transfers were seen in patients who lived far (>22.1 miles) from our hospital (32/69 (46%)) versus near our hospital (10/68 (15%)). For every mile boys lived from our hospital, there was no difference (adjusted odds ratio (OR)=0.98 (0.96, 1.00), p=0.10) in the likelihood of receiving an orchiectomy versus an orchiopexy when adjusting for age, symptom duration, and degrees of torsion.

Conclusions: Our study determined that neither distance from our hospital nor transfer from an outlying hospital affected the orchiectomy rate. An expedited medical evaluation and surgery offer the best prognosis for salvaging the testes.

## Introduction

With an incidence of 3.8 per 100,000 males <18 years annually, testicular torsion represents a pediatric surgical emergency requiring accurate and timely diagnosis to prevent testicular loss [1-4]. Presenting as sudden onset unilateral scrotal pain, this condition occurs when the spermatic cord rotates, which curtails testicular blood flow. Testicular pain greater than four-eight hours in duration is strongly associated with permanent ischemic damage [1,3-6]. Within the first six hours of symptom onset, there is a 90-100% chance of testicular salvage, which plunges to 0-10% by 12-24 hours [7]. Prompt surgery offers the best prospect of salvaging the testes. Despite timely surgical intervention, approximately 39-71% of boys will undergo an orchiectomy [1,3,8]. Testicular torsion is also associated with reduced fertility, testicular hormonal dysfunction, and psychological trauma [9]. 

 Several features have been reported that may delay the diagnosis of testicular torsion, which increases the probability that an orchiectomy will be performed, including isolated abdominal pain, a lack of complete genitourinary evaluation, scrotal ultrasound (US), delayed arrival at the Emergency Department (ED), and intellectual and developmental disabilities [4,5,10-14]. We have previously reported that testicular and epididymal heterogeneity as well as a thickened scrotal wall on Doppler scrotal US are associated with testicular demise in patients with testicular torsion, while testicular heterogeneity and scrotal wall thickening are more likely to occur with a longer symptom duration [13]. With a sensitivity of 89-100% and a specificity of 69-99%, an US is readily available, noninvasive, and has a low cost [13]. A patient’s distance from the hospital where he will be undergoing treatment for testicular torsion and transfer from an outside facility are two additional components that may impact whether the testis is saved [2,6,8,9,15-17]. However, the extant literature varies in importance of these factors predicting whether a patient will undergo an orchiectomy. It has been reported that the time from torsion onset to surgery is the most important element that influences the survival of the affected testis [8]. 

 The present study focuses on whether patient distance from our hospital and transfer from an outlying hospital affect numerous clinical and surgical characteristics associated with testicular torsion such as patient age, duration and side of symptoms, testicular pain, absent cremasteric reflex, type of surgery (orchiectomy versus orchiopexy), high-riding testis, and degree of testicular torsion. 

## Materials and methods

Under an Institutional Review Board-approved protocol and conforming to the Declaration of Helsinki, we identified male children and adolescents aged 1-18 years with testicular torsion between January 1, 2015 and December 31, 2020. The patients’ distance from our hospital and whether they were transferred from an outlying hospital were emphasized. A pediatric urologist acquired a medical history and conducted a concentrated genitourinary physical examination in our institution’s emergency department (ED). All patients underwent a Doppler scrotal US. Several characteristics were obtained including the patients’ age, duration and side of symptoms, testicular pain, absent cremasteric reflex, high-riding testis, type of surgery (orchiectomy versus orchiopexy), and degree of testicular torsion.

Statistical analysis

Counts (%) and median (interquartile range (IQR)) for each metric are summarized into two characteristic tables where boys (N=140) were bifurcated into two groupings: boys who lived near our hospital (N=69) versus far from our hospital (N=68) (Table 1) and transfer from an outlying hospital to our hospital (N=42) versus no transfer (N=98) (Table 2).

**Table 1:** Effect of distance from our hospital on characteristics of testicular torsion (January 1, 2015-December 31, 2020)

Characteristics	Overall N = 140	Far from Our Hospital N = 69	Near Our Hospital N = 68	P-Value
Age at time of surgery	14.0 (12.7, 15.3)	13.9 (12.6, 14.9)	14.3 (12.8, 15.6)	0.191
Duration of symptoms (hours)	10.0 (5.0, 48.0)	8.0 (5.0, 22.0)	12.0 (5.0, 48.0)	0.142
Duration of symptoms ≤6 hours	100 (71)	54 (78)	45 (66)	0.130
Duration of symptoms ≤12 hours	82 (59)	46 (67)	35 (51)	0.083
Duration of symptoms ≤24 hours	57 (41)	30 (43)	26 (38)	0.603
Transfer = Yes	42 (30)	32 (46)	10 (15)	<0.001
Testicular pain = Yes	136 (97)	68 (99)	65 (96)	0.366
Absent cremasteric reflex = Yes	42 (30)	18 (26)	23 (34)	0.355
High-riding testis = Yes	43 (32)	25 (37)	17 (26)	0.198
Surgery = orchiectomy	56 (40)	24 (35)	31 (46)	0.225
Degrees of torsion	360.0 (360.0, 720.0)	360.0 (180.0, 540.0)	450.0 (360.0, 720.0)	0.233
Duration of symptoms Very short (≤6 hours)	57 (41)	30 (43)	26 (38)	0.254
Short (≤12 hours)	25 (18)	16 (23)	9 (13)	
Medium (≤24 hours)	18 (13)	8 (12)	10 (15)	
Long (>24 hours)	40 (29)	15 (22)	23 (34)	
Side of torsed testis				0.124
Both	2 (1)	1 (1)	1 (1)	
Left	78 (56)	44 (64)	33 (49)	
Right	60 (43)	24 (35)	34 (50)	

**Table 2:** Effect of hospital transfer on characteristics of testicular torsion (January 1, 2015-December 31, 2020)

Characteristics	Overall N = 140	Not Transferred N = 98	Transferred N = 42	P-Value
Age at time of surgery	14.0 (12.7, 15.3)	14.1 (12.6, 15.5)	13.8 (12.8, 14.6)	0.229
Duration of symptoms (hours)	10.0 (5.0, 48.0)	10.5 (5.0, 48.0)	8.5 (5.0, 19.0)	0.157
Duration of symptoms ≤6 hours	57 (41)	38 (39)	19 (45)	0.574
Duration of symptoms ≤12 hours	82 (59)	54 (55)	28 (67)	0.262
Duration of symptoms ≤24 hours	1009 (71)	66 (67)	34 (81)	0.152
Testicular pain = Yes	136 (97)	95 (97)	41 (98)	1.000
Absent cremasteric reflex = Yes	42 (30)	30 (31)	12 (29)	0.844
High-riding testis = Yes	43 (32)	27 (28)	16 (41)	0.156
Surgery = orchiectomy	56 (40)	41 (42)	15 (36)	0.574
Degrees of torsion	360.0 (360.0, 720.0)	450.0 (360.0, 720.0)	360.0 (90.0, 540.0)	0.143
Duration of symptoms Very short (≤6 hours)	57 (41)	38 (39)	19 (45)	0.415
Short (≤12 hours)	25 (18)	16 (16)	9 (21)	
Medium (≤24 hours)	18 (13)	12 (12)	6 (14)	
Long (>24 hours)	40 (29)	32 (33)	8 (19)	
Side of torsed testis				0.794
Both	2 (1)	1 (1)	1 (1)	
Left	78 (56)	55 (56)	23 (55)	
Right	60 (43)	42 (43)	18 (43)	

The median split of the distance was calculated as 22.1 miles. Therefore, boys who lived within 22.1 miles of our hospital were considered near our hospital and those who lived more than 22.1 miles away from our hospital were characterized as far from our hospital. The Fisher's Exact Test compared categorical variables between groups, while the Wilcoxon Rank Sum Test contrasted the ordinal variables. Box and whisker plots juxtaposed distance distributions between surgery types. Multivariable logistic regression determined the adjusted odds ratio of miles from our hospital for boys who underwent an orchiectomy versus an orchiopexy. Odds ratios were adjusted by the best fitting controlling variables. Model building strategy were based on lowest Akaike information criterion (AIC), residual plots, and percentage change in beta coefficient of the puberty status variable. All analyses were performed using R software version 4.0.3 [18].

## Results

**Figure 1:**
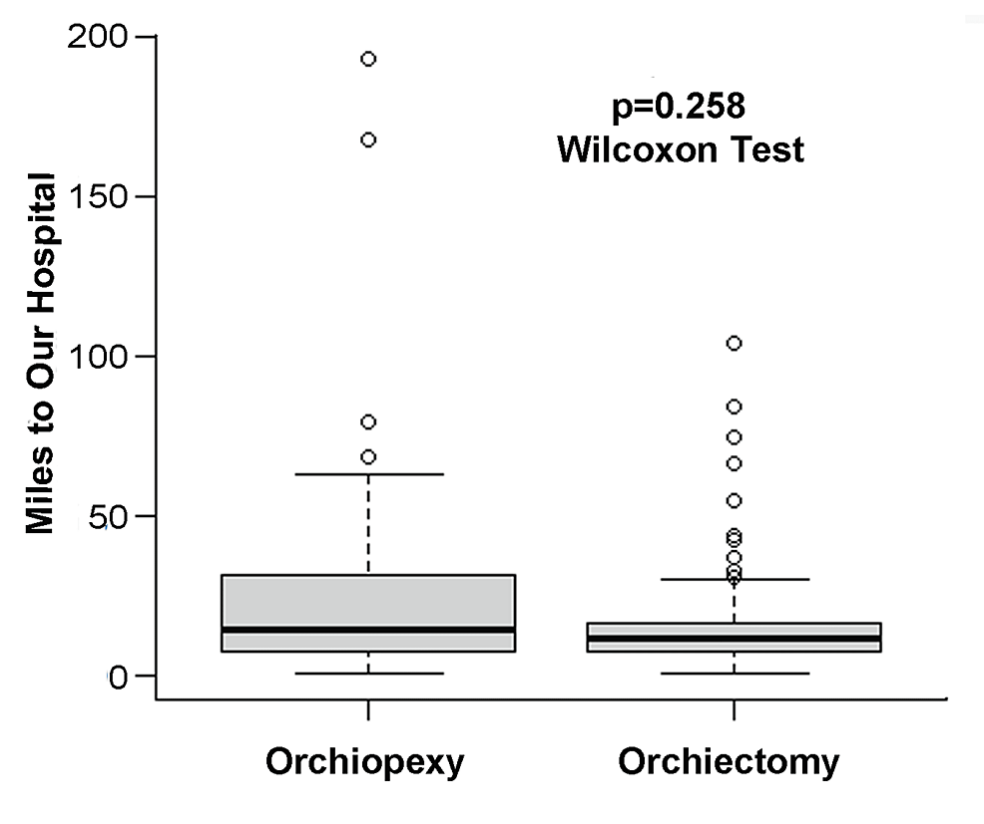
Distance from our hospital comparing boys who underwent an orchiectomy versus an orchiopexy

The number of miles from the patients’ residences to our hospital ranged between 0.7 and 193 miles (median: 22.1 miles). The patients’ distance from our hospital was not significantly different between boys who underwent an orchiectomy versus an orchiopexy (p=0.258) (Figure 1).

**Figure 2:**
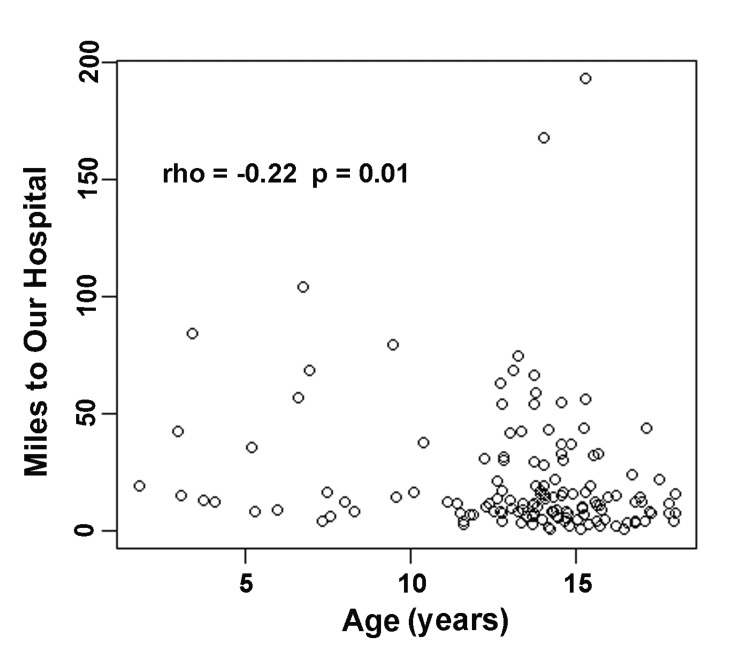
Distance from our hospital in relation to patient age at surgery

However, the number of miles from our hospital was negatively correlated to patient age at surgery (rho=-0.22, p=0.01) (Figure 2).

Boys with a shorter duration of symptoms (≤12 hours) tended to live further from our hospital (>22.1 miles) compared to those with a longer symptom duration (far=46/69 (67%) versus near=35/68 (51%), p=0.083). 

Transfer from an outlying hospital was not significantly different between boys who underwent an orchiectomy versus an orchiopexy (p=0.574) (Table 3).

**Table 3:** Transfer from an outlying hospital in relation to surgery type (orchiectomy versus orchiopexy)

	Transfer from an Outlying Hospital			
Type of Surgery	No	Yes	Total	P-Value
Orchiopexy	57 (68%)	27 (32%)	84 (100%)	0.574
Orchiectomy	41 (73%)	15 (27%)	56 (100%)	

Significantly more transfers (p<0.001) were seen in patients who lived far [>22.1 miles] from our hospital [32/69; 46%] versus near our hospital (10/68; 15%) (Tables 1,2). The odds of undergoing an orchiectomy were less likely for transferred patients regardless of cutoff for duration of symptoms (>6hrs (odds ratio (OR)=0.61 (0.20, 1.84), p=0.32), >12hrs (OR=0.54 (0.12, 2.52), p=0.48), and >24hrs (OR=0.40 (0.05, 3.25), p=0.35)).

Multivariable logistic regression

For every mile the boys lived from our hospital, there was no difference in the likelihood of receiving an orchiectomy versus an orchiopexy when adjusting for age, symptom duration, and degrees of torsion (adjusted OR=0.98 (0.96, 1.00), p=0.10).

## Discussion

Few studies have addressed the association between testicular torsion and distance from the hospital where treatment is performed and transfer from an outside hospital [2,6,8,9,15-17]. Romao and colleagues’ study of 1,713 patients with testicular torsion reported that distance traveled and transfer were not associated with outcomes [17]. In Kwenda and colleagues’ systematic review of 2,564 patients in nine retrospective studies of testicular torsion (532 transferred and 2,032 direct), transfer status did not have a significant effect on torsion outcomes [2]. However, of the patients with testicular torsion who presented within 24 hours of symptom onset, those who were transferred to another facility for management were more likely to undergo an orchiectomy than those treated at their presenting Institution. In Bayne and colleagues’ study of 97 boys with testicular torsion, the orchiectomy rate in patients who were versus were not transferred to the ED was 47.8 versus 68.9% (p=0.07) [8]. The mean transfer delay was 1 hour 15 minutes longer in the orchiectomy group (p=0.01). Multivariate analysis confirmed that symptom duration and distance from the hospital were the strongest predictors of orchiectomy. Similarly, in Preece and colleagues’ study of 125 patients with testicular torsion, transferred patients had twice the rate of testicular loss as those not transferred (30.4% versus 15.2%, p=0.129) [16]. Transferring a patient to a pediatric surgical center delays treatment and potentially puts the patient at an increased risk of testicular loss. Additionally, patients transferred over 30 miles had over 2.5 times the rate of testicular loss than those not transferred (42.8% versus 15.2%, p=0.029). Although there are many confounding factors in the management of testicular torsion, such as time since the onset of first symptoms, delay in seeking healthcare, and delay in a referral to urologic centers, distance from the treating hospital also plays an important role. The published data is not conclusive about the management of testicular torsion and, therefore, we wanted to add our experience in a state where there is a significant distance to the children’s hospital. Yiee and colleagues reported that hospital transfer was associated with orchiectomy on univariate but not multivariate analysis. However, this study included males of all ages and not just children and adolescents [9]. 

While it may seem that distance from our hospital and transfer from an outlying hospital may increase the orchiectomy rate due to an inability to salvage an infarcted testes, many studies have reported that these factors do not play a significant role in the delay of care. The time elapsed at home and with ED waiting and processing may be more important in determining surgery type [2]. In Romao and colleagues’ study, a longer time in the ED was associated with higher rates of testicular loss [17]. These authors reported that time spent in the ED awaiting diagnosis and treatment had a significant negative impact on the orchiectomy rates.

Our findings concur with certain studies in the literature reporting that distance from our hospital and transfer from an outside hospital did not contribute to whether a boy undergoes an orchiectomy versus an orchiopexy. There was also no difference in the likelihood of receiving an orchiectomy versus an orchiopexy for every mile boys lived from our hospital. However, more transfers were noted in patients who lived far from our hospital versus near our hospital. Our findings that distance and transfer from rural areas of our state did not change the orchiectomy rate are encouraging. They suggest that there was a high level of suspicion of testicular torsion and that boys were identified and transferred in a timely manner without an impact from either distance or transfer status. 

Strengths and limitations 

Our six-year study of testicular torsion highlighting the impact of distance from our hospital and transfer from an outlying hospital reveals that these factors do not influence the orchiectomy rate. Boys were transferred to our children’s hospital and were not treated in outlying facilities. Another strength of our study is that only one group of pediatric urologists treated boys with testicular torsion. Additionally, we assessed numerous metrics associated with testicular torsion. Limitations of the present work include its retrospective nature and relatively small number of patients. 

## Conclusions

While several key confounding factors play an important role in the management of testicular torsion, such as time since initial symptoms, severity of illness, and imaging findings of cases with orchiectomy versus orchiopexy, the distance from the treating hospital should be taken into consideration if the child presents with a prolonged duration of testicular pain and there is a lengthy transfer to the treating hospital. We sought to determine whether patients with testicular torsion who were primarily teenagers should be transferred to a children’s hospital with the associated potential risk of higher rates of orchiectomies or be treated at the facility of the initial patient presentation. Our study demonstrated that neither distance from our hospital nor transfer from an outlying hospital affected the number of orchiectomies. Physicians should be alert to the symptoms associated with testicular torsion. A prompt medical evaluation offers the best prognosis for salvaging the testes. 
